# Intraoperative collaboration between surgeons and endoscopists who performed previous endoscopic ultrasound in laparoscopic ultrasound-guided pancreatic tumor enucleation

**DOI:** 10.1055/a-2550-3975

**Published:** 2025-03-20

**Authors:** Kosuke Maehara, Satoshi Okubo, Kazuki Hirano, Daisuke Hattori, Yoshiki Sato, Rikako Koyama, Tsunao Imamura

**Affiliations:** 1 13600 Department of Gastroenterology, Toranomon Hospital, Minato-ku, Japan; 2 13600 Department of Gastroenterological Surgery, Toranomon Hospital, Minato-ku, Japan; 3 Okinaka Memorial Institute for Medical Research, Tokyo, Japan

Recent advancements in imaging technology and endoscopic ultrasound-guided fine-needle aspiration (EUS-FNA) have led to increased detection of small pancreatic tumors, enhancing the opportunities for surgical resection.

Laparoscopic pancreatic tumor enucleation is a suitable minimally invasive treatment, especially for tumors that usually have limited invasion into surrounding tissues, such as pancreatic neuroendocrine neoplasms and solid pseudopapillary neoplasms smaller than 10 mm 1 2 3 . However, the small size of these tumors makes them difficult to visualize with laparoscopic ultrasound, potentially complicating tumor localization and leading to over-extraction. At our institution, we have implemented a strategy to improve the accuracy of intraoperative tumor localization by having the endoscopist who performed the preoperative EUS also conduct the laparoscopic ultrasound during surgery. This approach may allow for more precise tumor identification and resection, avoiding excessive tissue removal while preserving the minimally invasive nature of the tumor enucleation.

We present the case of a 67-year-old man with a suspected pancreatic body cyst identified on an abdominal ultrasound, which was later confirmed on EUS as a 4-mm hypoechoic mass ( Fig. 1 ). EUS-FNA ( Fig. 2 ) revealed a diagnosis of pancreatic neuroendocrine neoplasm (G1). The patient opted for minimally invasive surgery, and laparoscopic enucleation was performed.

**Fig. 1:**
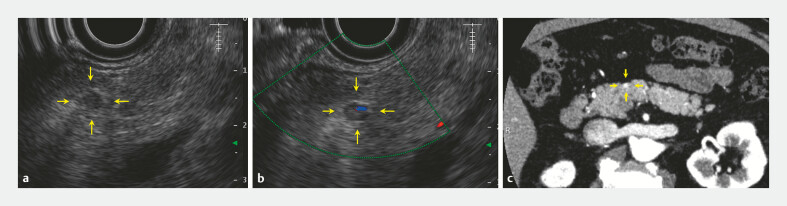
Initial imaging of the small pancreatic tumor (arrows). **a, b** Endoscopic ultrasound. **c** Contrast-enhanced computed tomography.

**Fig. 2:**
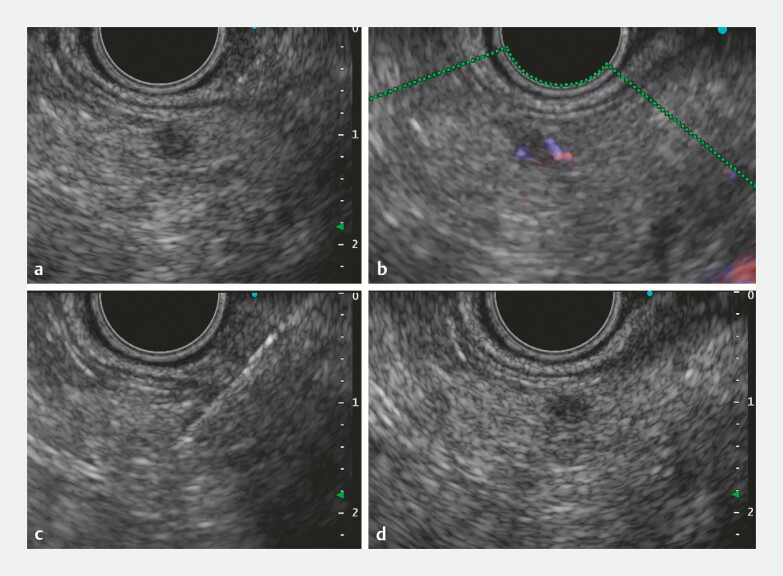
Endoscopic ultrasound-guided fine-needle aspiration of 4-mm pancreatic tumor. **a** B mode. **b** Color Doppler. **c** Fine-needle aspiration. **d** Confirming tumor recognition after puncture.

Initially, the surgeon’s laparoscopic ultrasound (ARIETTA 60; Hitachi, Ltd., Tokyo, Japan) using linear-array transducer failed to visualize the tumor, leading to multiple unsuccessful extractions. The endoscopist who had performed the preoperative EUS then took over the laparoscopic ultrasound, successfully delineating the tumor ( Fig. 3 , Video 1 ). The surgeon confirmed the location, and tumor enucleation was completed successfully ( Fig. 4 ).

**Fig. 3:**
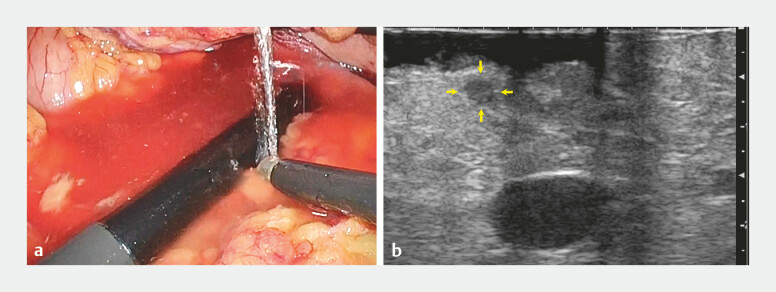
Detecting the small pancreatic tumor using laparoscopic ultrasound by collaboration between surgeons and endoscopists. **a** Laparoscopic ultrasound probe with water immersion. **b** Laparoscopic ultrasound imaging (yellow arrows, small pancreatic tumor).

**Fig. 4:**
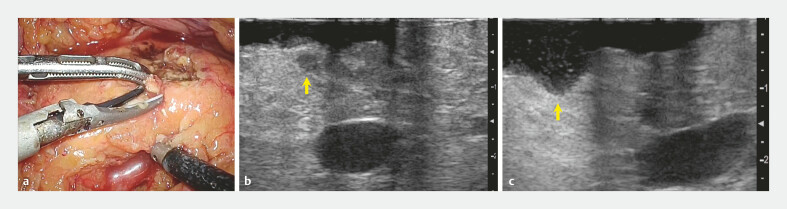
Enucleation of the small pancreatic tumor (arrow). **a** Laparoscopic image. **b, c** Comparison of laparoscopic ultrasound imaging before ( **b** ) and after ( **c** ) enucleation.

Intraoperative collaboration between surgeons and endoscopists in the use of laparoscopic ultrasound for pancreatic tumor enucleation. ESU, endoscopic ultrasound; MPD, main pancreatic duct; SMV, superior mesenteric vein.Video 1

Postoperatively, the patient had no complications and showed no recurrence at the 9-month follow-up.

This case highlights the importance of intraoperative collaboration between surgeons and endoscopists to ensure accurate localization and successful outcomes in minimally invasive pancreatic surgery.

Endoscopy_UCTN_Code_TTT_1AS_2AD

## Notes

Endoscopy E-Videos https://eref.thieme.de/e-videos
*E-Videos* is an open access online section of the journal *Endoscopy* , reporting on interesting cases and new techniques in gastroenterological endoscopy. All papers include a high-quality video and are published with a Creative Commons CC-BY license. Endoscopy E-Videos qualify for HINARI discounts and waivers and eligibility is automatically checked during the submission process. We grant 100% waivers to articles whose corresponding authors are based in Group A countries and 50% waivers to those who are based in Group B countries as classified by Research4Life (see: https://www.research4life.org/access/eligibility/ ). This section has its own submission website at https://mc.manuscriptcentral.com/e-videos .
